# Perception of scientific uncertainty about climate change and associations with anxiety and maternal–fetal attachment among pregnant women

**DOI:** 10.3389/fpubh.2026.1930658

**Published:** 2026-09-04

**Authors:** Congcong Zhao, Xianghong Cheng, Li Liu, Qi Zhao, Zhenzhen Wang, Zheyuan Zhao, Shengnan He, Jialei Feng

**Affiliations:** 1 Department of Obstetrics, Henan Provincial People’s Hospital, Henan Provincial Intelligent Nursing and Transformation Engineering Research Center, Henan Provincial Key Medicine Laboratory of Nursing, Zhengzhou University People’s Hospital, Zhengzhou, China; 2 Department of Obstetrics, Gynecology and Reproductive Medicine Center, Peking University First Hospital, Beijing, China

**Keywords:** climate change, maternal–fetal attachment, mediation analysis, perception of scientific uncertainty, pregnant women, prenatal anxiety

## Abstract

**Introduction:**

Perception of scientific uncertainty about climate change (PSUCC) may contribute to emotional distress and impede maternal psychological adaptation during pregnancy. However, evidence in pregnant women remains limited. This study examined the level and predictors of PSUCC, as well as its association with prenatal anxiety and maternal–fetal attachment.

**Methods:**

Convenience sampling method was used to select pregnant women from three tertiary hospitals in Henan Province, China, between October and December 2025. A total of 537 pregnant women completed questionnaires assessing demographic and obstetric characteristics, PSUCC, prenatal anxiety, and maternal–fetal attachment. Multiple linear regression, Pearson correlation analysis, and structural equation modeling (SEM) were used for data analysis.

**Results:**

The mean scores of PSUCC, prenatal anxiety, and maternal–fetal attachment were 30.09 ± 7.63, 11.09 ± 4.56, and 48.02 ± 14.83, respectively. PSUCC was positively correlated with prenatal anxiety (*r* = 0.679, *p* < 0.001) and negatively correlated with maternal–fetal attachment (*r* = −0.672, *p* < 0.001). SEM showed that PSUCC was directly associated with increased prenatal anxiety and reduced maternal–fetal attachment (attachment strength and attachment quality). In addition, prenatal anxiety partially mediated the relationship between PSUCC and maternal–fetal attachment. Overall, higher PSUCC was associated with greater prenatal anxiety and weaker maternal–fetal attachment among pregnant women.

**Conclusion:**

The findings indicating that PSUCC may be a psychosocial stressor associated with anxiety and maternal–fetal attachment among Chinese pregnant women. The results support the inclusion of climate-related risk communication and psychological support in routine prenatal care to promote maternal psychological adaptation.

## 1 Introduction

Climate change, defined as long-term changes in temperature and weather patterns, has become one of the most critical global challenges of the 21st century, with consequences for environmental systems and human health (1). Growing evidence suggests that these impacts extend beyond physical health and impose psychological burdens (2). Among vulnerable populations, pregnant women are particularly susceptible, owing to physiological changes and increased emotional sensitivity during the perinatal period (3). Beyond direct environmental exposures, increasing attention has been paid to the psychological perception of climate change. Perception of scientific uncertainty about climate change (PSUCC) refers to individuals’ subjective perception of ambiguity regarding climate-related information, scientific evidence, and potential consequences (4). Such perceived uncertainty may function as a chronic psychological stressor, eliciting negative emotional responses such as anxiety, worry, and fear. Nevertheless, research on this topic in pregnant populations remains limited.

Prenatal anxiety is a common mental health concern during pregnancy, with reported prevalence in China ranging from 14.95 to 45.92% (5, 6). Elevated levels of prenatal anxiety have been associated with adverse maternal and fetal outcomes, including increased risks of preterm birth, low birth weight, and disrupted functional connectivity in the fetal brain (7). Maternal–fetal attachment refers to the emotional bond that develops between a pregnant woman and her fetus. It is an indicator of maternal psychological wellbeing and a key factor in the development of early mother-infant relationships (8). Previous research has indicated that higher prenatal anxiety is associated with weaker maternal–fetal attachment, suggesting that emotional distress during pregnancy may disrupt early bonding processes (3).

Although climate change is increasingly recognized as a determinant of mental health (9), few studies have examined how PSUCC affects psychological wellbeing among pregnant women (10–12). Previous studies have primarily focused on emotional responses to climate change (10, 11). Notably, Amin et al. (10) reported significant associations among emotional responses to climate change, antenatal anxiety, and maternal–fetal attachment in pregnant women. However, the potential influence of PSUCC on prenatal anxiety and maternal–fetal attachment remains insufficiently understood, particularly in China (10). Furthermore, the psychological mechanisms underlying these associations have yet to be fully clarified (10, 11). Based on these findings, this study examined whether prenatal anxiety mediates the relationship between PSUCC and maternal–fetal attachment may provide further insight into the psychological impact of PSUCC during pregnancy.

This study aims to (1) assess the level of PSUCC and its associated factors among pregnant women, (2) examine its associations with prenatal anxiety and maternal–fetal attachment, and (3) explore the potential mediating role of prenatal anxiety in these relationships. The findings are expected to inform the development of tailored psychological interventions and climate-related health education for pregnant women.

## 2 Materials and methods

### 2.1 Design

This cross-sectional study was based on data collected between October and December 2025 from an anonymous internal assessment of clinical quality and health management conducted in three tertiary hospitals in Zhengzhou, Henan Province, China. The original assessment did not involve clinical intervention, collection of identifiable information, or biological specimens. The secondary use of the anonymized dataset for research and academic publication was approved by the Medical Ethics Committee of Henan Provincial People’s Hospital (Approval No. 2026-LS-017). Written informed consent was obtained from all participants before data collection. All procedures were performed in accordance with the Declaration of Helsinki and relevant ethical guidelines. The reporting of this study followed the Strengthening the Reporting of Observational Studies in Epidemiology (STROBE) Statement (13).

Pregnant women attending the perinatal clinic were recruited using convenience sampling. Eligible participants were those who (1) were aged 18 years or older, (2) had a gestational age of at least 12 weeks, and (3) provided written informed consent. Women were excluded if they (1) had severe cognitive impairment or communication difficulties that prevented them from completing the questionnaires, or (2) had a clinically diagnosed psychiatric disorder.

The required sample size was estimated based on the recommendation that studies using multivariable regression analysis should include 10–20 participants per independent variable (14). After accounting for an invalid rate of approximately 10%, the estimated sample size ranged from 374 to 748 participants. A total of 548 questionnaires were distributed and all were returned (yielding a 100% response rate). After data screening, responses were excluded if completion time was less than 200 s (the minimum time considered sufficient for careful reading and response) or if all items were answered with identical responses. Ultimately, 537 valid questionnaires were included in the final analysis, representing a data validity of 97.99%.

### 2.2 Measures

#### 2.2.1 Sociodemographic and pregnancy-related characteristics

Sociodemographic and obstetric information was collected using a self-designed questionnaire. The questionnaire comprised 15 items covering age, residence, marital status, educational level, occupation, monthly per capita household income, gestational age, gravidity, parity, abnormal pregnancy history, planned pregnancy, pregnancy complications, average daily sleep duration during pregnancy, attention to climate change-related topics, and self-reported exposure to extreme weather events attributed to climate change.

#### 2.2.2 PSUCC

The perception of scientific uncertainty about climate change scale (PSUCC) was developed by Visschers (12) and translated into Chinese by Qu (4). The scale consists of eight items rated on a 6-point Likert scale (1 = strongly disagree, 2 = disagree, 3 = somewhat disagree, 4 = somewhat agree, 5 = agree, 6 = strongly agree). Higher total scores indicate a greater perception of scientific uncertainty about climate change. The scale comprises three dimensions: measurement uncertainty (items 1, 2, 3), uncertainty about future outcomes (items 4, 5, 6), and ambiguity (items 7, 8). Previous validation of the Chinese version supported its three-factor structure through confirmatory factor analysis (CFA), with acceptable model fit indices (*χ*^2^/df = 3.934, SRMR = 0.044, GFI = 0.949, NFI = 0.955, IFI = 0.966, TLI = 0.944, CFI = 0.966, RMSEA = 0.092). The Cronbach’s *α* for the total scale was 0.893 in the validation study, and for the three dimensions, the values were 0.894, 0.700, and 0.751, respectively. In this study, the Cronbach’s *α* for the total scale was 0.931.

#### 2.2.3 Prenatal anxiety

The Generalized Anxiety Disorder-7 Scale (GAD-7) was developed by Spitzer et al. (15) and translated into Chinese by He et al. (16). This instrument is used for both screening and assessing the severity of anxiety disorders. The scale consists of seven items, each scored from 0 (not at all) to 3 (nearly every day). The total score is classified into four severity levels: minimal (0–4), mild (5–9), moderate (10–14), and severe (15–21). Previous studies have demonstrated good internal consistency, with a Cronbach’s *α* of 0.912. In this study, the Cronbach’s *α* was 0.911.

#### 2.2.4 Maternal antenatal attachment

The Maternal Antenatal Attachment Scale (MAAS) was developed by Condon (17), and translated into Chinese by Nie et al. (18). This instrument is utilized to assess the emotional bond between a mother and her fetus during pregnancy (17). The scale consists of 19 items rated on a 5-point Likert scale, with higher total scores indicating stronger maternal–fetal attachment. The scale includes two dimensions: attachment quality (items 3, 6, 7, 9, 10, 11, 12, 13, 15, 16, and 19) and attachment strength (items 1, 2, 4, 5, 8, 14, 17, 18). The Cronbach’s *α* was 0.77 for the total scale and 0.69 and 0.62 for the two dimensions, respectively. In this study, the Cronbach’s was 0.902 for the total scale and 0.936 and 0.889 for the two dimensions, respectively.

### 2.3 Quality control

Before data collection, all investigators received standardized training to ensure consistency in the study procedures. Eligible participants accessed the online questionnaire by scanning a QR code with their mobile devices after receiving standardized instructions. The questionnaire was designed with mandatory response settings to minimize missing data and was configured to allow only one submission per device. After data collection, questionnaires with excessively short completion times or uniform response patterns were excluded. The exported dataset was then reviewed for completeness and accuracy before statistical analyses.

### 2.4 Statistical analysis

All statistical analyses were conducted using IBM SPSS Statistics 26.0, and structural equation modeling (SEM) was performed using IBM SPSS Amos 26.0. Continuous variables are presented as mean ± standard deviation (SD), and categorical variables as frequencies and percentages. Independent-samples *t*-tests were used to compare continuous variables between two groups, and one-way analysis of variance (ANOVA) was used for comparisons among three or more groups.

Differences in PSUCC scores across demographic and obstetric subgroups were examined using independent-samples *t*-tests or one-way ANOVA, as appropriate. Pearson correlation analyses were performed to examine correlations among PSUCC, prenatal anxiety, and maternal–fetal attachment.

Variables that were significantly associated with PSUCC in univariate analyses (*p* < 0.05), together with theoretically relevant covariates identified from previous studies (19), were included in a multiple linear regression model Table 1. Age was added as a continuous variable using its original value. Categorical variables were transformed into dummy variables before entry. Categorical variables were coded with 0 as the reference category and 1 as the comparator. For education level, dummy variables were created with a bachelor’s degree as the reference, and for monthly per capita household income, the reference category was set at <3,000 CNY.

**Table 1:** Coding scheme for variables included in the multiple linear regression model.

Variable	Coding	Reference category
Age	Continuous (years)	–
Residence	0 = urban 1 = rural	Urban (0)
Abnormal pregnancy history	0 = no 1 = yes	No (0)
Planned pregnancy	0 = planned 1 = unplanned	Planned (0)
Pregnancy complications	0 = no 1 = yes	No (0)
Attention to climate change related topics	0 = yes 1 = no	Yes (0)
Education level	1 = junior high or below 2 = high school/technical secondary 3 = junior college 4 = bachelor’s degree 5 = master’s or above	Bachelor’s degree (4)
Monthly per capita household income (CNY)	1 = <3,000 2 = 3,000–4,999 3 = 5,000–9,999 4 = ≥10,000	<3,000 (1)

Multicollinearity among variables was evaluated using variance inflation factors (VIFs), within a VIF < 5 indicating no substantial multicollinearity.

Harman’s single-factor test was used to examine common method bias (CMB), in which all 34 items from the PSUCC, GAD-7, and MAAS were entered into an unrotated principal component analysis (PCA).

To examine whether prenatal anxiety mediated the relationship between PSUCC and maternal–fetal attachment, SEM was conducted using an overall model for total maternal–fetal attachment, as well as separate subdimension models for strength and quality of attachment. Model fit was evaluated using standard goodness-of-fit indices with commonly used criteria: *χ*^2^/df < 5, RMSEA <0.08, and GFI, AGFI, CFI, TLI > 0.90. The significance of the indirect mediating effect was tested using bias-corrected bootstrapping with 5,000 resamples. A mediating effect was considered significant if its 95% confidence interval (CI) did not include zero.

All statistical tests were two-tailed, and *p* < 0.05 was considered statistically significant.

## 3 Results

### 3.1 Participant characteristics and univariate analysis of PSUCC

A total of 537 pregnant women were included in the analysis. The median age was 32 years. Univariate analyses revealed that PSUCC differed significantly by age, residence, education level, abnormal pregnancy history, planned pregnancy, pregnancy complications, and attention to climate change related topics (*p* < 0.05, Table 2).

**Table 2:** Basiline characteristics and univariate analysis of PSUCC (*N* = 537).

Information	*N* (%)	PSUCC	*F*/*t*	*p*
Age				
≤34	381 (70.90)	29.55 ± 7.56	6.619^(1)^	0.001
35–39	138 (25.70)	30.86 ± 7.49		
≥40	18 (3.35)	35.67 ± 7.86		
Residence				
Urban	400 (74.49)	31.20 ± 7.16	5.614^(2)^	0.000
Rural area	137 (25.51)	26.85 ± 8.05		
Marital status				
Married	510 (95.00)	30.15 ± 7.63	0.985^(1)^	0.374
Divorced	9 (1.68)	26.56 ± 7.26		
Never married	18 (3.35)	30.00 ± 7.81		
Educational level				
Junior high school and below	20 (3.72)	27.25 ± 12.04	6.987^(1)^	0.000
High school/technical secondary school	95 (17.69)	26.93 ± 8.51		
Junior college	152 (28.31)	29.61 ± 6.00		
Bachelor’s degree	193 (35.94)	31.36 ± 7.09		
Master’s degree or above	77 (14.34)	32.49 ± 7.83		
Occupation				
Staff	189 (35.20)	30.76 ± 7.43	0.995^(1)^	0.420
Civil servant	51 (9.50)	30.02 ± 7.11		
Professional and technical staff	134 (24.95)	29.82 ± 7.35		
Farmer	3 (0.56)	29.67 ± 6.66		
Self-employed people	76 (14.15)	28.54 ± 8.82		
Unemployed	84 (15.64)	30.48 ± 7.69		
Monthly per capita household income (CNY)				
<3,000	107 (19.93)	30.31 ± 7.70	0.781^(1)^	0.505
3,000–4,999	150 (27.93)	29.69 ± 7.55		
5,000–9,999	183 (34.08)	30.66 ± 7.70		
≧10,000	97 (18.06)	29.39 ± 7.56		
Gestional age				
<37	421 (78.40)	30.24 ± 7.62	0.898^(2)^	0.369
≥37	116 (21.60)	29.53 ± 7.66		
Gravidity				
1	159 (29.6)	30.10 ± 8.02	0.519^(1)^	0.595
2	213 (39.7)	27.73 ± 7.20		
≥3	165 (30.7)	30.54 ± 7.80		
Parity				
0	293 (54.60)	30.05 ± 7.63	0.977^(1)^	0.403
1	213 (39.66)	30.26 ± 7.37		
2	23 (4.28)	30.52 ± 9.57		
≥3	8 (1.49)	25.63 ± 8.19		
Abnormal pregnancy history				
No	499 (92.92)	29.81 ± 7.65	−3.619^(2)^	0.001
Yes	38 (7.08)	33.74 ± 6.34		
Planned pregnancy				
Yes	354 (65.92)	29.28 ± 7.70	−3.467^(2)^	0.001
No	183 (34.08)	31.66 ± 7.26		
Pregnancy complications				
Yes	203 (37.80)	28.33 ± 7.16	−4.231^(2)^	0.000
No	334 (62.20)	31.16 ± 7.72		
Average daily sleep duration during pregnancy				
≦6 h	55 (10.24)	30.44 ± 7.28	0.132^(1)^	0.876
7–9 h	428 (79.70)	30.10 ± 7.69		
≧10 h	54 (10.06)	29.69 ± 7.59		
Attention to climate change related topics				
Yes	170 (31.66)	31.28 ± 7.47	2.465^(2)^	0.014
No	367 (68.34)	29.54 ± 7.65		
Self-reported exposure to extreme weather events attributed to climate change				
Yes	370 (68.90)	30.38 ± 7.55	1.295^(2)^	0.196
No	167 (30.10)	29.46 ± 7.78		

PSUCC = Perception of scientific uncertainty about climate change. Superscripts (1) and (2) represent *F*-statistics from one-way ANOVA and *t*-statistics from independent-samples *t*-tests, respectively.

### 3.2 Descriptive statistics and correlation analysis

In this study, the mean total score for the PSUCC was 30.09 ± 7.63, and the mean GAD-7 score was 11.09 ± 4.56. The mean MAAS score was 48.02 ± 14.83, with 21.13 ± 8.23 for strength of attachment and 26.88 ± 10.7 for quality of attachment. As shown in Table 3, PSUCC was positively correlated with prenatal anxiety (*p* < 0.001) and negatively correlated with maternal–fetal attachment (*p* < 0.001).

**Table 3:** Correlations analysis among the variables.

Variable	1	2	3	4	5
PSUCC (1)	1.000	–	–	–	–
Prenatal anxiety (2)	0.679	1.000	–	–	–
Maternal antenatal attachment (3)	−0.672	−0.657	1.000	–	–
Strength of attachment (4)	−0.419	−0.392	0.787	1.000	–
Quality of attachment (5)	−0.629	−0.633	0.763	0.202	1.000

PSUCC = perception of scientific uncertainty about climate change. Correlation is significant at the 0.01 level (2-tailed).

### 3.3 Multiple linear regression analysis

Multiple linear regression showed that the model was significant (adjusted R^2^ = 0.185, *F* = 10.370, *p* < 0.001) (Table 4). VIF ranged from 1.012 to 2.187, indicating no substantial multicollinearity. After adjusting for all covariates, PSUCC was positively associated with age (*β* = 0.087, *p* < 0.05), abnormal pregnancy history (*β* = 0.098, *p* < 0.05), planned pregnancy (*β* = 0.120, *p* < 0.05), pregnancy complications (*β* = 0.117, *p* < 0.05), and attention to climate change-related topics (*β* = 0.152, *p* < 0.001). Residence was negatively associated with PSUCC (*β* = −0.306, *p* < 0.001). Compared with a bachelor’s degree, only the high school/ technical secondary school group had significantly lower PSUCC scores (*β* = −0.164, *p* < 0.001). Other education levels were not significantly associated with PSUCC (*p* > 0.05). For monthly per capita household income, compared to the <3,000 CNY reference group, the 3,000–4,999 CNY (*β* = −0.178, *p* < 0.05) and ≥10,000 CNY (*β* = −0.133, *p* < 0.05) groups had significantly lower PSUCC scores, while the 5,000–9,999 CNY group did not differ significantly (*p* > 0.05).

**Table 4:** Multiple linear regression analysis.

Variable	*B*	Std. error	*β*	*t*	*p*	Tol.	VIF
Constant	26.843	2.333	–	11.506	0.000	–	–
Age	0.143	0.064	0.087	2.229	0.026	0.988	1.012
Residence	−5.357	0.810	−0.306	−6.612	0.000	0.708	1.412
Abnormal pregnancy history	2.901	1.170	0.098	2.479	0.013	0.981	1.020
Planned pregnancy	1.926	0.641	0.120	3.005	0.003	0.957	1.045
Pregnancy complications	1.834	0.636	0.117	2.883	0.004	0.929	1.077
Attention to climate change related topics	2.492	0.702	0.152	3.552	0.000	0.832	1.202
Educational level = Junior high school and below	−3.058	1.629	−0.076	−1.877	0.061	0.929	1.077
Educational level = High school/technical secondary school	−3.278	0.886	−0.164	−3.701	0.000	0.773	1.293
Educational level = Junior college	−1.027	0.766	−0.061	−1.341	0.180	0.742	1.347
Educational level = Master’s degree or above	1.231	0.936	0.057	1.315	0.189	0.820	1.219
Monthly per capita household income (CNY) = 3,000–4,999	−3.016	0.979	−0.178	−3.080	0.002	0.458	2.186
Monthly per capita household income (CNY) = 5,000–9,999	−1.586	0.927	−0.099	−1.711	0.088	0.457	2.187
Monthly per capita household income (CNY) = ≧10,000	−2.640	1.082	−0.133	−2.441	0.015	0.510	1.960

*R*^2^ = 0.205, adjusted *R*^2^ = 0.185, *F* = 10.370, *p* < 0.001. *B* = unstandardized coefficient, Std. Error = standard error; *β* = standardized coefficient; *t* = *t*-statistic, Tol. = Tolerance, VIF = variance inflation factor. All VIFs were below 5 (ranging from 1.012 to 2.187), indicating no significant multicollinearity in the model.

### 3.4 Mediation analysis

**Figure 1:**
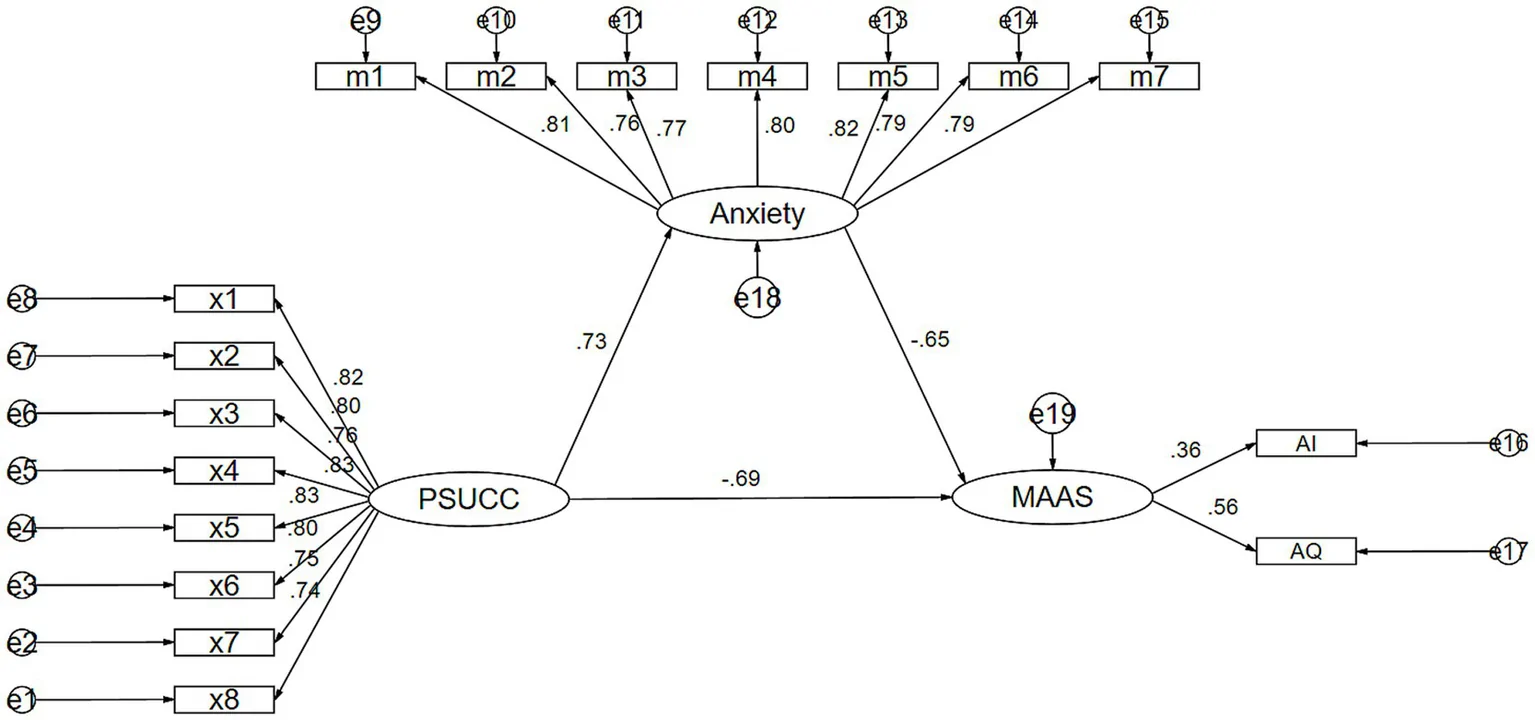
Mediation model 1. PSUCC = perception of scientific uncertainty about climate change. Anxiety = prenatal anxiety. MAAS = maternal antenatal attachment. AI = attachment strength. AQ = attachment quality.

A primary mediation model was first constructed with PSUCC as the independent variable, prenatal anxiety as the mediator, and maternal–fetal attachment as the dependent variable (Figure 1). The model exhibited good fit to the data: *χ*^2^/df = 1.866, RMSEA = 0.040, GFI = 0.954, AGFI = 0.939, CFI = 0.983, and TLI = 0.981. Path analysis showed significant structural relationships among the variables (Table 5). PSUCC had a significant positive effect on prenatal anxiety (*β* = 0.730, *p* < 0.05). Both PSUCC (*β* = −0.692, *p* < 0.05) and prenatal anxiety (*β* = −0.651, *p* < 0.05) directly and negatively predicted maternal–fetal attachment.

**Table 5:** Standardized path coefficients and factor loading for SEM 1.

Path	Std. estimate (*β*)
Anxiety ← PSUCC	0.730
MAAS ← Anxiety	−0.651
MAAS ← PSUCC	−0.692
x8 ← PSUCC	0.737
x7 ← PSUCC	0.750
x6 ← PSUCC	0.805
x5 ← PSUCC	0.831
x4 ← PSUCC	0.831
x3 ← PSUCC	0.756
x2 ← PSUCC	0.802
x1 ← PSUCC	0.822
m1 ← Anxiety	0.809
m2 ← Anxiety	0.758
m3 ← Anxiety	0.766
m4 ← Anxiety	0.803
m5 ← Anxiety	0.816
m6 ← Anxiety	0.789
m7 ← Anxiety	0.794
AI ← MAAS	0.361
AQ ← MAAS	0.561

PSUCC = Perception of scientific uncertainty about climate change. Anxiety = Prenatal anxiety. MAAS = Maternal antenatal attachment. AI = Attachment strength. AQ = Attachment quality. All standardized coefficients are statistically significant at *p* < 0.05.

Bias-corrected bootstrapping with 5,000 resamples was performed to test the mediating effect. The 95% CI did not include zero, indicating a significant partial mediating effect of prenatal anxiety (Table 6). The total effect was −1.167 (95% CI: −1.444 to −1.015, *p* < 0.05). The direct effect was −0.691 (95% CI: −0.948 to −0.479, *p* < 0.05), accounting for 59.21% of the total effect. The indirect effect was −0.476 (95% CI: −0.695 to −0.295, *p* < 0.05), accounting for 40.79% of the total effect.

**Table 6:** Mediation analysis results.

Effect type	Path	Effect	Standard Error	95% CI		Proportion (%)	*p*
				Lower	Upper		
Maternal antenatal attachment							
Direct effect	PSUCC–maternal antenatal attachment	−0.691	0.118	−0.948	−0.479	59.21	0.001
Indirect effect	PSUCC–prenatal anxiety–maternal antenatal attachment	−0.476	0.130	−0.695	−0.295	40.79	0.001
Total effect	PSUCC–maternal antenatal attachment	−1.167	0.108	−1.444	−1.015	100.00	0.001
Attachment strength							
Direct effect	PSUCC–attachment strength	−0.301	0.079	−0.450	−0.150	66.89	0.001
Indirect effect	PSUCC–prenatal anxiety–attachment strength	−0.149	0.057	−0.265	−0.041	33.11	0.010
Total effect	PSUCC–attachment strength	−0.450	0.046	−0.533	−0.356	100.00	0.001
Attachment quality							
Direct effect	PSUCC–attachment strength quality	−0.380	0.074	−0.531	−0.245	56.80	0.001
Indirect effect	PSUCC–prenatal anxiety–attachment quality	−0.289	0.053	−0.393	−0.188	43.20	0.001
Total effect	PSUCC–attachment quality	−0.669	0.036	−0.736	−0.598	100.00	0.001

PSUCC = Perception of scientific uncertainty about climate change.

**Figure 2:**
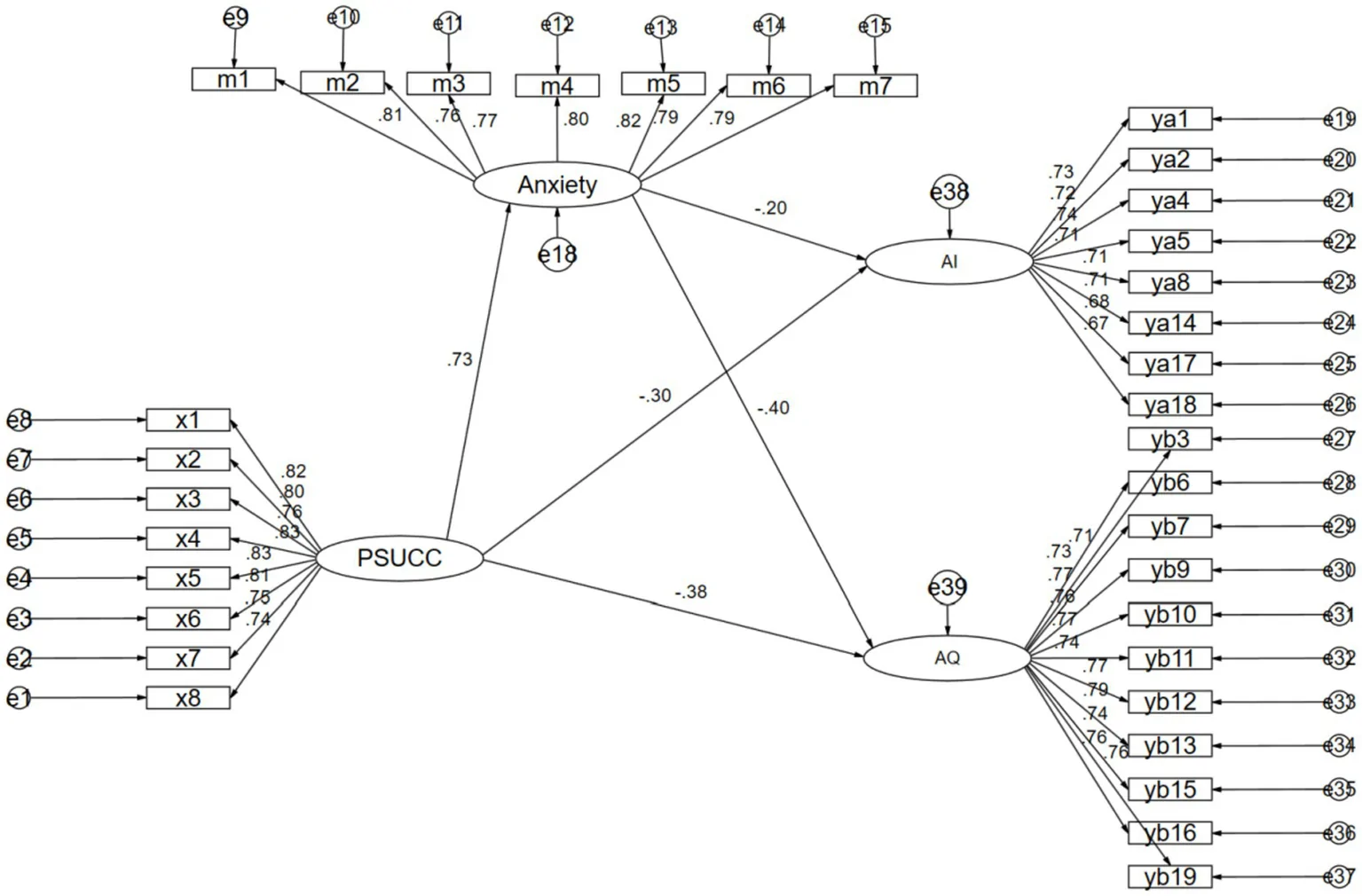
Mediation model 2. PSUCC = perception of scientific uncertainty about climate change. Anxiety = prenatal anxiety. AI = attachment strength. AQ = attachment quality. All standardized coefficients are statistically significant at *p* < 0.05.

Two separate mediation models were then constructed, one for strength of attachment and one for quality of attachment (Figure 2). Both models showed good fit to the data: *χ*^2^/df = 1.318, RMSEA = 0.024, GFI = 0.932, AGFI = 0.923, CFI = 0.985, and TLI = 0.984. PSUCC positively predicted prenatal anxiety (*β* = 0.730, *p* < 0.05) (Table 7). In both models, PSUCC negatively predicted attachment strength (*β* = −0.302, *p* < 0.05) and attachment quality (*β* = −0.380, *p* < 0.05). Prenatal anxiety also negatively predicted strength of attachment (*β* = −0.204, *p* < 0.05) and quality of attachment (*β* = −0.395, *p* < 0.05).

**Table 7:** Standardized path coefficients and factor loading for SEM 2.

Path	Std. estimate (*β*)
Anxiety ← PSUCC	0.730
AI ← Anxiety	−0.204
AQ ← Anxiety	−0.395
AQ ← PSUCC	−0.380
AI ← PSUCC	−0.302
x8 ← PSUCC	0.736
x7 ← PSUCC	0.750
x6 ← PSUCC	0.805
x5 ← PSUCC	0.831
x4 ← PSUCC	0.831
x3 ← PSUCC	0.756
x2 ← PSUCC	0.803
x1 ← PSUCC	0.822
m1 ← Anxiety	0.809
m2 ← Anxiety	0.758
m3 ← Anxiety	0.766
m4 ← Anxiety	0.804
m5 ← Anxiety	0.816
m6 ← Anxiety	0.789
m7 ← Anxiety	0.794
ya1 ← AI	0.727
ya2 ← AI	0.720
ya4 ← AI	0.737
ya5 ← AI	0.705
ya8 ← AI	0.709
ya14 ← AI	0.715
ya17 ← AI	0.678
ya18 ← AI	0.674
yb3 ← AQ	0.713
yb6 ← AQ	0.733
yb7 ← AQ	0.775
yb9 ← AQ	0.764
yb10 ← AQ	0.770
yb11 ← AQ	0.743
yb12 ← AQ	0.769
yb13 ← AQ	0.789
yb15 ← AQ	0.738
yb16 ← AQ	0.755
yb19 ← AQ	0.760

PSUCC = Perception of scientific uncertainty about climate change. Anxiety = Prenatal anxiety. AI = Attachment strength. AQ = Attachment quality. All standardized coefficients are statistically significant at *p* < 0.05.

Bootstrap results confirmed significant partial mediating effects in both models. For strength of attachment, the total effect of PSUCC was −0.450 (95% CI: −0.533 to −0.356, *p* < 0.05), with a direct effect of −0.301 (95% CI: −0.450 to −0.150, *p* < 0.05) accounting for 66.89% of the total effect, and an indirect effect of −0.149 (95% CI: −0.265 to −0.041, *p* < 0.05) accounting for 33.11%. For attachment quality, the total effect was −0.669 (95% CI: −0.736 to −0.598, *p* < 0.05), with a direct effect of −0.380 (95% CI: −0.531 to −0.245, *p* < 0.05) accounting for 56.80% of the total effect, and an indirect effect of −0.289 (95% CI: −0.393 to −0.188, *p* < 0.05) accounting for 43.20%.

### 3.5 Common method bias assessment

Harman’s single-factor test was conducted to evaluate potential common method bias. The first factor explained 40.492% of the total variance, indicating that no single factor accounted for the majority of the total variance. Thus, common method bias was unlikely to have significantly influenced the results.

## 4 Discussion

To our knowledge, this is the first study to investigate the relationships among PSUCC, prenatal anxiety, and maternal–fetal attachment in Chinese pregnant women. Four major findings were obtained. First, Chinese pregnant women reported above-midpoint levels of PSUCC. Second, multivariable analysis showed that age, abnormal pregnancy history, planned pregnancy, pregnancy complications, and attention to climate change topics were positively associated with PSUCC, while residence, the education level of high school/ technical secondary school (compared with a bachelor’s degree), and monthly per capita household income of 3,000–4,999 CNY and ≥10,000 CNY (compared to the <3,000 CNY) was negatively associated. Third, PSUCC was positively associated with prenatal anxiety and negatively associated with maternal–fetal attachment, including both attachment strength and attachment quality. Finally, prenatal anxiety partially mediated the relationship between PSUCC and maternal–fetal attachment, as well as its two dimensions, suggesting a potential psychological pathway through which climate change uncertainty may influence maternal psychological adaptation during pregnancy.

The above-midpoint level of PSUCC observed suggests that climate change has become a notable concern among pregnant women. Unlike many traditional health threats, climate change is characterized by complex scientific evidence, uncertain future consequences, and difficulties in predicting individual-level impacts (20). Previous studies have suggested that uncertainty surrounding environmental risks may be more psychologically distressing than clearly defined threats, as uncertainty reduces individuals’ sense of predictability and control (21). During pregnancy, women may become particularly vigilant to pregnancy-related threats, with concerns often focused on fetal wellbeing, maternal health, and adverse pregnancy outcomes (22, 23). Consequently, PSUCC may represent not merely a perception of an environmental issue, but a perceived threat to maternal and fetal health.

Several sociodemographic and pregnancy-related factors were associated with PSUCC in this study. Older pregnant women and those living in urban areas had higher PSUCC scores. These differences may reflect differences in exposure to climate-related information, personal experiences with climate change, and perceived health risks. Education level and monthly per capita household income did not exhibit a consistent pattern across categories, suggesting that socioeconomic status alone may not explain differences in perceptions of climate-related uncertainty. Individual experiences and perceptions may also influence PSUCC. Pregnancy-related characteristics were also associated with PSUCC. Women with a history of abnormal pregnancy, pregnancy complications, or a planned pregnancy may pay more attention to maternal and fetal health and may therefore be more sensitive to potential environmental risks. In addition, greater attention to climate change was associated with higher PSUCC scores. This finding may suggest that people who actively follow climate-related issues are more aware of the uncertainties surrounding climate science.

Our findings demonstrated a positive association between PSUCC and anxiety. This is consistent with previous work on climate-related anxiety, which identifies uncertainty about climate change as a contributor to psychological distress, anxiety symptoms, and reduced emotional wellbeing (2, 21). Studies in adolescents, young adults, and general populations have shown that concerns about climate change can elicit persistent worry, fear about the future, and feelings of helplessness (24, 25). For women, heightened concern about the impacts of climate change has also been associated with reduced intentions to childbearing (11). By extending this line of research from reproductive decision-making into the prenatal period, this study identifies climate change uncertainty as a significant source of anxiety during pregnancy.

Importantly, our findings suggest that the psychological impact of climate change may arise not only from attention to climate change but also from uncertainty about scientific knowledge and future outcomes. People tend to feel more distress when they cannot accurately assess the likelihood, severity, or controllability of potential risks (26). PSUCC may therefore undermine the confidence of pregnant women in their ability to anticipate and manage environmental threats, which in turn may contribute to anxiety (27). Our findings extend the concept of climate-related anxiety to the prenatal context by suggesting that scientific uncertainty itself may act as a psychosocial stressor during pregnancy. Given that anxiety during pregnancy has been associated with adverse maternal and neonatal outcomes, these results further support the growing recognition of climate change as an emerging mental health challenge.

This study also found that PSUCC was negatively associated with maternal–fetal attachment, including both attachment quality and attachment strength. Maternal–fetal attachment is an important aspect of maternal psychological adaptation and has been associated with maternal wellbeing, prenatal health behaviors, and mother-infant relationships. Although previous studies have shown that climate-related psychological stressors are associated with greater anxiety and psychological distress, and that higher prenatal anxiety is associated with weaker maternal–fetal attachment (28–30), limited evidence exists regarding the relationship between PSUCC and maternal psychological adaptation during pregnancy. Therefore, our findings extending literature by identifying PSUCC as an underexplored factor associated with maternal–fetal attachment. Several mechanisms may explain this relationship. According to attachment theory, the development of emotional bonds requires psychological availability, emotional security, and positive expectations about the future (31, 32). When pregnant women experience persistent uncertainty about environmental conditions and potential threats to fetal health, psychological resources may become increasingly focused on threat monitoring and risk management. As a result, fewer emotional and cognitive resources may remain available for positive engagement with the fetus. Persistent uncertainty may encourage pregnant women to adopt cognitive and emotional avoidance to cope with perceived threats, thereby reducing emotional investment in the fetus. Although such protective disengagement may serve as an adaptive short-term coping response, it may weaken the development of maternal–fetal attachment over time (33). This interpretation is consistent with growing evidence that psychological distress, stress, and anxiety during pregnancy are associated with reduced maternal–fetal attachment and impaired emotional adaptation to pregnancy (30, 34). A particularly important finding is that prenatal anxiety partially mediated the relationship between PSUCC and maternal–fetal attachment. To our knowledge, few studies have explored the psychological mechanisms through which climate change uncertainty to emotional outcomes in pregnant women. Existing research on climate and health has focused on general mental health, such as anxiety, depression, and psychological distress (35, 36). By including maternal–fetal attachment as an outcome, this study broadens our understanding of how climate change uncertainty may affect emotional adaptation during pregnancy.

The mediating effect of prenatal anxiety can be further explained by Protection Motivation Theory, which posits that individuals evaluate both the severity of a perceived threat and their ability to cope with it before developing emotional and behavioral responses (37). When pregnant women perceive climate change as a potential threat to their own health or fetal health, while also experiencing uncertainty about scientific evidence and effective coping strategies, they may feel more vulnerable and less capable of managing the situation. This perception of reduced control may, in turn, increase prenatal anxiety (25). Persistent anxiety may subsequently impair emotional regulation, heighten attention to potential threats, and reduce positive emotional engagement with the fetus, ultimately weakening maternal–fetal attachment (10, 19). Therefore, prenatal anxiety may represent an important psychological mechanism through which uncertainty about climate change may influence emotional wellbeing during pregnancy. These findings suggest that interventions should focus on reducing anxiety arising from uncertainty, thereby promoting maternal psychological adaptation during pregnancy.

In summary, these findings add to the literature on climate change and mental health by demonstrating that the psychological consequences of climate change may arise not only from direct exposure to environmental changes or from general concern about climate change, but also from perceived scientific uncertainty (26).

By identifying prenatal anxiety as a partial mediator between PSUCC and maternal–fetal attachment, this study provides a novel framework for understanding how cognitive responses to climate change influence emotional adaptation during pregnancy. These findings broaden current understanding of psychological vulnerability during pregnancy and suggest that PSUCC may represent an important but underexplored pathway through which climate change affects psychological wellbeing during pregnancy.

### 4.1 Practical implications

Given the limited evidence on PSUCC among pregnant women in China, these findings have implications for clinical practice and for policies on maternal health. The findings indicate that PSUCC may be a psychosocial risk factor during pregnancy and therefore requires greater attention in prenatal care. Prenatal care could be expanded to include education on climate and health, clear communication about climate-related risks, anxiety screening, and psychological support. These measures may help reduce distress related to uncertainty, ease anxiety during pregnancy, and support the development of maternal–fetal attachment. As climate change continues to accelerate, addressing psychological responses to climate change within maternal healthcare may strengthen the resilience of prenatal care systems and improve health outcomes for both mothers and children.

Several limitations should be considered. First, longitudinal studies are needed to better understand the temporal relationships among PSUCC, prenatal anxiety, and maternal–fetal attachment, as the cross-sectional design of this study does not allow causal inferences. Second, all data were collected using self-reported questionnaires at a single time point, which may have introduced some bias. However, Harman’s single-factor test indicated that common method bias was unlikely to have had a major impact on the results. Third, participants were recruited from three tertiary hospitals in Zhengzhou, China, which may limit the applicability of the findings to other populations and regions. Fourth, this study did not consider some potentially important factors, such as social support, coping strategies, resilience, and exposure to environmental changes. Future studies should include these factors and use longitudinal designs to further explore how climate-related perceptions, psychological responses, and maternal health outcomes are connected. Finally, although the Chinese version of the PSUCC scale showed good reliability in previous validation studies and in the present study, its adaptation has mainly been reported in a master’s thesis rather than in a peer-reviewed journal. Further validation studies involving more diverse populations are therefore needed.

## 5 Conclusion

This study showed that Chinese pregnant women reported above-midpoint levels of PSUCC. Higher PSUCC was associated with greater prenatal anxiety and weaker maternal–fetal attachment. In addition, prenatal anxiety partially mediated the relationship between PSUCC and maternal–fetal attachment. These findings suggest that scientific uncertainty about climate change may be a psychosocial stressor during pregnancy. As climate change increasingly influences population health, clinicians and researchers should pay more attention to how pregnant women perceive environmental uncertainty and how they respond psychologically to climate-related risks. Incorporating education on climate and health, effective communication about climate-related risks, psychological support, and anxiety management into prenatal care may help reduce distress caused by uncertainty and support the development of maternal–fetal attachment. Overall, this study expands current understanding of how climate change affects psychological wellbeing during pregnancy and provides a foundation for future research and for developing interventions in maternal health care that are responsive to climate change.

## Data Availability

The original contributions presented in the study are included in the article/supplementary material, further inquiries can be directed to the corresponding author/s.
